# Factors affecting smoking initiation and cessation among adult smokers in Fiji: A qualitative study

**DOI:** 10.18332/tid/143027

**Published:** 2021-12-06

**Authors:** Masoud Mohammadnezhad, Mondha Kengganpanich

**Affiliations:** 1 School of Public Health and Primary Care, Fiji National University, Suva, Fiji; 2 Department of Health Education and Behavioral Sciences, Faculty of Public Health, Mahidol University, Bangkok, Thailand

**Keywords:** determinants, smoking initiation, smoking cessation, qualitative study, Fiji

## Abstract

**INTRODUCTION:**

Smoking as a public health challenge is globally considered the main risk factor of many non-communicable diseases (NCDs). Knowing factors contributing to smoking commencement and cessation is the necessary step to develop prevention strategies to combat this issue. To date, no study has been conducted in Fiji, therefore this study aimed to explore the reasons adult smokers initiate smoking and cessation in Fiji.

**METHODS:**

A qualitative study was conducted among 35 current smokers who were interviewed between 1 May and 31 July 2020 in Suva, Fiji. Three health centers were chosen randomly to collect data and purposive sampling was applied to reach study participants. A semi-structured, open-ended questionnaire was used to guide the interviews. The content of in-depth interviews was transcribed and data were analyzed using content and thematic analysis.

**RESULTS:**

The results of this study showed that most of the participants were male (57%), I-taukei (77%), single (54%), had attained tertiary education level (69%), were of Christian religion (77%), and unemployed (63%). Two main themes were identified including: ‘factors affecting smoking initiation’ and ‘factors affecting smoking cessation’. ‘Peer pressure’, ‘smoking myth’, ‘smoking as a fun’, ‘unpleasant event in life’ and ‘smoking establishes friendships’ were factors affecting initiation of smoking; while ‘knowledge on smoking harms’, ‘financial constraints’, ‘desire to improve health’, ‘constant request from family members’, ‘desire to save time’, ‘religious factors’ and ‘cultural factors’, were factors affecting smoking cessation among smokers.

**CONCLUSIONS:**

This study highlights the main factors affecting smoking among adult smokers in Fiji. Considering these factors in future health planning will help policy makers and decision makers to develop tailored interventions to combat this health issue.

## INTRODUCTION

Smoking is considered the main risk factor for many non-communicable diseases (NCDs) such as cardiovascular disease, lung cancer etc., and is also the major cause of preventable deaths, globally^1,2^. Death from tobacco smoking constitutes 11.5% of deaths worldwide and is the main reason for hospitalizations, even more than alcohol and drugs combined^3^. Spaulding et al.^4^ revealed that in 2015, 25% of men and 5% of women smoked, globally. The smoking prevalence among the Pacific people, along with its illness related burden, remains high in other countries, especially in New Zealand. In the Fijian context, there is a decline in daily tobacco smoking in both sexes and all ethnicities over the past 30 years, however, prevalence remains high in men at around 27%^5^. The results of the NCD Steps Survey that was conducted in 2011 showed that the prevalence of smoking was higher among younger adults aged 25–34 years. It was also more prevalent in the iTaukei Fijian community, as 23% of iTaukei women were smokers compared to 2.1% in Indo-Fijian women^6^.

Henceforth, improving smoking cessation programs is important and understanding the perspectives of smokers on factors affecting initiation and smoking cessation is also important as it can help form the planning of smoking cessation programs to meet the needs of smokers when they intend to quit^7^. Some of the common perspectives that are often associated with a smoker initiating smoking include: smoking as a coping mechanism for social problems they face; being curious of what it is like to smoke; and smoking being a social norm and widely practiced^7,8^. Further, having a strong influence from smoking friends; thinking that smoking makes the mind more relaxed; smoking makes the person appear as more grown up and independent from parents; and smoking helps people to forget their problems^9-11^. On the other hand, some of the perceptions that are commonly known in the literature about factors that encourage smoking cessation include: bans on indoor smoking; restricting advertising and selling of tobacco; support of family and friends; and spiritual support^12-14^. To date, there has not been a study conducted in Fiji on factors contributing to smoking initiation and cessation, therefore this study aimed to explore the reasons for adults becoming smokers and the factors affecting smoking initiation and cessation in Suva, Fiji.

## METHODS

### Study design and setting

This study applied a qualitative design to explore factors affecting smoking amongst current adult smokers who attended three public health centers in Suva, Fiji, between 1 May and 31 July 2020. Nuffield, Samabula, and Valelevu were chosen randomly among seven health centers. In this study, public clinics were chosen as smokers were accessible for a long time and facilities to conduct interview were also available.

### Population and sample

All smokers attending the three selected health centers were considered as the study population. Patients or family members accompanying the patient who were smokers during the study period or had smoked at least 10 cigarettes per day and had high nicotine addiction, measured by the Fagerström test, both males and females, and self-identified as Fijian, were included in the study. Those who were nonsmokers were excluded and smokers who had mental instability or were not willing to participate in the study, were excluded. A purposive sampling was used to choose participants and data saturation was reached after interviewing 35 participants.

### Data collection tools

A semi-structured, open-ended questionnaire was used to guide the interviews. The questionnaire was developed based on the literature and research questions. The questionnaire had two sections including demographic questions and 7 open-ended questions to understand the factors affecting smoking initiation and cessation.

### Study procedures

All potential participants were informed about the aim and procedure of the study one week before starting the interviews through flyers and announcements provided by the health centers. Those who met the study criteria were asked to read an information sheet and sign a consent form. A trained research assistant conducted all the interviews after scheduling face-toface interviews that were feasible for both interviewer and interviewee, in a privacy room in each heath center. Each interview took 30–40 minutes and was recorded.

### Data management and analysis

All interviews were transcribed by the research assistant and triangulation was checked by the other researchers. Thematic analysis was used to analyze the data. To do that, each transcript was read and re-read, by the research assistant and the main researcher, to find the sentences which were most frequently repeated in each interview. Using a codebook, codes were identified and similar codes were merged to determine subthemes; similar subthemes were combined to identify common themes^15^. If the identified codes were not similar there was a discussion between two coders to resolve the difference.

### Study rigor

In a qualitative study, it is important to ensure rigorousness and trustworthiness. In this study, trustworthiness was based on four criteria: credibility, dependability, confirmability and transferability^16,17^. For the credibility, all participants were informed about the aim and study procedure before involving them in the study, both verbally and using an information sheet. All participants were asked to sign a consent form. Interviews were conducted at a time and place that were suitable for both interviewer and interviewees. Essential information was provided to the research assistant in terms of conducting the interview and data collection. The interview tool was pilot tested before implementing it in the interview. All the interviews were recorded by a digital recorder and transcription performed on the same day of the interview. For dependability, transcriptions were read and re-read to correct all potential errors. Two independent coders read all the transcripts and identified codes separately and discussed the differences. For the confirmability, all the researchers discussed the identified codes, themes and subthemes, and confirmed them. Transferability was done using purposive sampling and by continuing interviews until data saturation was reached.

Permission to conduct interviews was obtained from the medical officers in charge of the selected health centers. All participants were informed about the aim and objectives of the study, the potential benefits, or harms and their right to participate in the study using an information sheet. Those who met the study criteria were asked to sign the consent form. Information sheet and consent form were provided in Fijian, Fiji Hindi and in English, to give freedom to participants to choose the version they were comfortable with. They were informed that participating in the study is completely voluntary and they could stop participation at any stage. Participants were kept anonymous and all information was confidential. After completing interviews, for the participants who had smokingrelated health issues, advice was provided and they were asked to visit a doctor.

## RESULTS

Table 1 gives information on the sociodemographic characteristics of the 35 study participants. Most participants were aged 18–24 years (49%), male (57%), iTaukei (77%), single (54%), had attained tertiary education level (69%), of Christian religion (77%), and were unemployed (63%).

**Table 1:** General characteristics of adult smoker participants in Suva, 2020 (N=35)

*Characteristics*	*n*	*%*
**Age** (years)		
18–24	17	49
25–29	6	17
30–34	6	17
≥35	6	17
**Gender**		
Male	20	57
Female	15	43
**Ethnicity**		
iTaukei	27	77
Fijian of Indian descent	7	20
Rotuman	1	3
**Marital status**		
Married	12	34
Single	19	54
Separated	3	9
Divorced	1	3
**Education level**		
Tertiary	24	69
Secondary	11	31
Primary	0	0
**Religion**		
Christian	27	77
Hindu	6	17
Muslim	1	33
Roman Catholic	1	
**Employment status**		
Unemployed	22	63
Employed	13	37

Two themes were identified after analyzing the data. They include ‘factors affecting smoking initiation’ and ‘factors affecting smoking cessation’. Each theme includes different subthemes that are presented in Table 2. To protect participants’ confidentiality, participants are presented as P1, P2, etc.

**Table 2:** Themes and subthemes identified from interviews among adult smoker participants in Suva, 2020 (N=35)

*Themes*	*Subthemes*
**Factors affecting smoking initiation**	1. Peer pressure
	2. Smoking myth
	3. Smoking as fun
	4. Unpleasant event in life
	5. Smoking establishes friendships
**Factors affecting smoking cessation**	1. Knowledge on smoking harms
	2. Financial constraints
	3. Desire to improve health
	4. Constant request from family members
	5. Desire to save time
	6. Religious factors
	7. Cultural factors

### Theme 1: Factors affecting smoking initiation

This theme has five subthemes including: ‘peer pressure’, ‘smoking myth’, ‘smoking as fun’, ‘unpleasant event in life’ and ‘smoking establishes friendships’.

#### Subtheme 1: Peer pressure

Most of the study participants mentioned that they started smoking because of peer pressure when they were a student:

*‘Um … back then I was occupied with studies in high school and I have uh … not that I have that much problem but it's just uh … I believe it was also because of peer pressure. Back in school um … the boarding life when someone smokes you would want to be in the system so I was smoking with them. Um … after a while, I thought that I won't be able to be addicted but uh … only God knows that I am now.’* (P34; 24 years, Male)

Another participant mentioned that:

*‘The main reason why I started smoking was through peer pressure when I saw my friends smoke back in high school, so they also invited me to join them, so that's when I started smoking.’* (P17; 32 years, Male)

They believed that smoking tobacco, like other substances, was common especially in the place they were living:

*‘… because we've been aahh. In the village. Yeah, we've been with them since child's hood, we like aahh get used to what we do so I eehh just uumm pressured by what they do in the village … like drinking and drinking grog and alcohol and even smoking’*. (P4; 43 years, Male)

Or the place they visit with friends:

*‘The first time I smoked uh…. I was amongst some of my friends and um … They invited me to go and have some beers and that's when I started smoking cigarettes. The first time I had it, I was coughing and coughing and then I finally did it.’* (P29; 38 years, Female)

#### Subtheme 2: Smoking myth

Most of the participants have also reasoned their smoking as a way of relieving stress:

*‘The reason for smoking is just to relax and sometimes after work or when I'm in conflict I just smoke so that I can relieve the stress. I find out that smoking is more relaxing than listening to other things.’* (P35; 21 years, Male)

Another participant stated:

*‘I don't know, aahh friends maybe because they've once tried it. For me personally I think it's true because whenever there's piles of work and I'm stressed out, aahh this smoke it really … releases stress, you know tension is just relieved.’* (P8; 19 years, Male)

#### Subtheme 3: Smoking as fun

Some of the participants stated that they started smoking because they perceived it as something fun to do:

*‘… We just do it for fun, we have come to a point where we become addicted to smoking and we cannot quit.’* ( P19; 48 years, Female )

Another participant stated:

*‘Personally, I believe that this time around aahh … it's, like one of my aunties is telling me, this is the time to enjoy yourself. Once you get onto the field you will never get this, this chance of life spend that is why I don't want to give up at this moment of time.’* (P8; 19 years, Male)

The role of social events, like graduation, was highlighted in starting smoking:

*‘The first time I smoke the first cigarette was … yeah this is among my graduation friends after the graduation … probably we ended up having a farewell party and so this is where it all started … Yeah! Yeah! It was just meant to be fun but it turned out to be really serious.’* (P27; 32, Male)

#### Subtheme 4: Unpleasant event in life

The majority of the participants stated that the reason for their smoking was they had a tragic experience at some point in their life and that smoking is an activity that helps them cope with the tragic moment:

*‘Started when I lost a family member, very close relative of mine, that's when I was, I’ve been hearing that smoking relieves stress and helps you to calm down, so I tried out smoking and every time I think of this family member of mine I turn to smoking.’* (P3; 20 years, Male)

Another participant mentioned:

*‘Uumm … because I was 20 years old when my dad passed away, that's the reason why I started smoking because I was really grieving for my father's death.’* (P10; 28 years, Female)

#### Subtheme 5: Smoking establishes friendships

Most of the participants stated that smoking helps them to establish friendships and be part of a group or circle of friends:

*‘Uumm … it was yeah, yeah, so that I can just fit in to the society with the groups, you know in order to fit in to groups you have to be like them, so that is why, basically I think it, yeah peer or something.’* (P8; 19 years, Male)

Another participant mentioned:

*‘… I think the main reason behind it was because of my peers and I think that if I don't smoke, I'm not part of that circle of friends. So this was back in school and when we did it, it was done without people knowing and when I said people these include my teachers, my mentors in school and of course my parents back at home.’* (P34; 24 years, Male)

A participant even mentioned that he regarded the person who shares a cigarette with him to be his true friend:

*‘You know for me friendship is something that I cherish and so when someone smokes with me … oh … He or she will be my really good friends.’* (P34; 24 years, Male)

Some participants mentioned that they usually share a cigarette with others during smoking and they call these practices as ‘peace-pipe’:

*‘Aahh … I just stop smoking for 3 months and start again because of peer pressure, mainly when I drink grog with my friends. Aahh and they ask me to join in peace-pipe.’* (P10; 28 years, Female)

Another participant stated:

*‘umm … because when I drink grog that is why I wanted to start again. Also, roaming around with my friends while they are smoking, I can smell it and so I wanted to start again.’* (P20; 25 years, Female)

### Theme 2: Factors affecting smoking cessation

This theme has seven subthemes including: ‘knowledge on smoking harms’, ‘financial constraints’, ‘desire to improve health’, ‘constant request from family members’, ‘desire to save time’, ‘religious factors’ and ‘cultural factors’.

#### Subtheme 1: Knowledge on smoking harms

Most of the participants acknowledged that smoking could damage their health and they know it well:

*‘I just don't know how to stop now … because now I am getting worried about my health … now every time I get a pain in my chest … I get afraid what if I am having cancer … or something like that.’* (P7; 25 years, Male)

Another participant mentioned:

*‘… because myself I really want to quit smoking so when they come and help me, it will be a big help for me um … because it can damage heart, damage lungs and show me some diagram to help me see what is bad about smoking, so it will be a big help for me.’* (P29; 38 years, Female )

They considered the warning information, as an image helps them to know about smoking harm:

*‘That is the whole reason and also the images. The images show that it can damage our lungs.’* (P28; 21 years, Female)

Some of the participants had even mentioned that they had witnessed firsthand the ravaging damage that smoking can do to their health.

*‘… ummmmm … sometimes I get sick and I usually think that this probably due to the fact that I smoke too much. I can really feel that it is due to smoking cigarettes. I think that's it.’* (P26; 32 years, Female)

A relative’s death helped them to consider smoking cessation:

*‘… also at first hand, I've seen relatives dying from lung cancers due to smoking because he was a heavy smoker. For me, I had always had that at the back of my mind, yes I will one day give up.’* ( P34; 24 years, Male )

#### Subtheme 2: Financial constraints

Most participants mentioned that financial constraints and the urge to save money were factors that motivated them to quit smoking:

*‘… aahh another good effect yes you may, mostly you will save money coz now the price of a single packet is $7.00 plus eh … so maybe if you, if I quit smoking I will save a lot of my money and also aahh … enjoying my food.’* ( P6; 23 years, Male )

Some mentioned that saving money and health benefits are enough for them to quit smoking:

*‘Yes, in terms of saving money, it will be useful for me. Well, money is just part of it but the health which cannot be bought by money, right? So that's a big benefit.’* (P27; 32 years, Male)

#### Subtheme 3: Desire to improve health

Most of the participants mentioned that smoking cessation improves health:

*‘Okay, umm so, umm. Basically, ahh it would improve my health, and umm my ahh dental health as well. So, yeah basically it is gonna improve my health.’* (P30; 32 years, Female)

They also believed that quitting smoking will help them to gain weight and improve their physical appearance:

*‘Yes! I think it will help us to gain some weight as I will eat well, and our physical appearance will improve too.’* (P18; 18 years, Male)

#### Subtheme 4: Constant request from family members

Most of the participants had also mentioned that they believed that the constant request from parents and other family members can help motivate them to quit smoking.

*‘… but I am trying my best because … I have been inspired by the people like my mum … my grandma who has never smoked completely… so I told them how do they do it … they leave it slowly … they cannot just leave it all of the sudden … I will just leave it all of a sudden …’* (P7; 25 years, Male)

#### Subtheme 5: Desire to save time

Some participants mentioned that quitting smoking helps them to minimize time wasted on unnecessary activities such as smoking:

*‘… and yes, because I can focus more on my schoolwork because usually when we go out smoking, we spend one, two hours, not just smoking but chit chat stuff. So yes it saves time.’* (P25; 20 years, Female)

#### Subtheme 6: Religious factors

Most of the participants mentioned that religion is a facilitating factor that helps smokers to pursue smoking cessation:

*‘… ahhhhh maybe just go to church and umm because ahhh going to church is that relationship with God is ummm clean so yes I think going to church would help me a lot (help quit smoking).’* ( P5; 21 years, Male )

They believe this can happen, especially during the observance of some special religious occasions:

*‘Yes, I adhere to it since its being implemented by the church, if not, something bad might happened to me. The church has really helped me a lot, not only during the taboo, but also by sharing the scriptures in the bible, really help me that time to quit smoking.’* (P17; 32 years, Male)

#### Subtheme 7: Cultural factors

Some of the participants mentioned that smoking cessation was considered as a group agreement for a period of time, so that it is a kind of respect to law or unwritten rule:

*‘In our village or … in our chief … we … together with the church they come up with a plan to control smoking and grog in the village revise the plans so that everyone in that village who are there or even away from there should follow this taboo by the tikina on grog, alcohol and cigarette … and that is like 2 weeks of every month so we never stop … so that people living there and the ones living here through my working living areas we don't smoke as a sign of respect to our tikina an church to be abide by the law.’* (P7; 25 years, Male)

In some situations, smoking is a taboo that should be considered:

*‘So according to our culture, the Fijian iTaukei aahh … when someone died aahh … specially in the family aahh we are supposed to make the taboo. That taboo, even for me it's like I stop smoking, because I am supposed to be engaged in the taboo since it's for my dad’s. I quitted smoking for 2 months.’* (P10; 28 years, Female)

Participants also stated that it is a custom in some villages that women are not allowed to smoke cigarettes:

*‘… my background or family background where I come from, it's a very religious background and traditional. In a traditional family, ladies or girls do not smoke. It's highly recommended that they are not. They are told, we are told Uhm … that it's a taboo in our family for ladies or young women to smoke. Yes, also in a sense that I'm still schooling and cannot buy a packet of smoke, we are told not to smoke.’* (P28; 21 years, Female)

## DISCUSSION

Smoking is a major issue globally, considered the main reason behind the prevalence of premature deaths. Countries worldwide are working hard to get evidence to help with evidence-based interventions to successfully combat it. The evidence from this qualitative study gives a picture of the factors affecting smoking initiation and smoking cessation among adult smokers in Suva, Fiji. The findings are similar to studies in other parts of world.

The findings support the claims of other studies that some of the factors affecting smoking initiation include peer pressure, misperception that smoking relieves stress (smoking myth) and that smoking is perceived as fun and is a pleasurable activity^18-20^. It is also worthwhile to note from this study that some respondents genuinely believe from their experience that smoking relieves stress and tension. Furthermore, our findings show that some people begin smoking as a way of coping with their loss or sadness, a way of establishing friendships with others and that smoking goes well with drinking kava and alcohol. This is similar to the findings from studies in other countries, which noted that smoking is associated with alcohol and drug consumption^21-26^. These findings could also be the main reason why Tomlinson^27,28^, mentioned that smoking is deeply integrated into the lifestyle of the vast majority of the Fijian people who like to socialize, when kava consumption is often widely practiced. This is vital information that could help inform health providers on the best approach and mechanisms of proactively addressing the prevalence of smoking in Fiji.

In terms of smoking cessation, this study also found some factors that affect smoking cessation including: requests from family members and friends and support to quit smoking^29^; health issues^30^; religious factors^31^; financial constraints^32^; health and fitness; and increased social awareness^33,34^. Nevertheless, it is noted that role of culture, such as the observance of a taboo during cultural events and occasions such as funerals, is a unique factor that helps facilitate the process of smoking cessation among smokers. Religious factors, such as the observance of the Lent season among Christians and EID among Muslims, have also been found in this study as an important reason for smokers to quit smoking. On the other hand, similar to other studies, it is noted that some of the barriers to smoking cessation found in this study include: strong influence from smoking friends and families^35^; nicotine addiction^36-38^; accessibility of cigarettes^39^; misunderstanding that quitting smoking can damage health; lack of support from social networks such as family and friends^39,40^; misperception and/or misunderstanding that accepting cigarettes from a friend is a token of friendship^41,42^; and the lack of willpower to refuse a cigarette when being offered by a smoking friend^39^.

The findings of this study provide valuable guidelines to public health professionals. They should adopt their training based on individual characteristics of smokers by considering the differences in socioeconomic status as well as cultural background. For Fiji, as people have different ethnicities, culture, and languages, it is important to apply community based smoking cessation programs based on the real needs of smokers^43^. Schools should be actively involved in anti-smoking campaigns, to prevent smoking at an early age. Developing antismoking policies and taxation should be considered as a priority at the governmental level.

### Limitations

Although, this is the first qualitative study about smoking in Fiji, this study was conducted only among adult smokers. Conducting a study among all age groups may give more valuable information regarding the factors affecting smoking in Fiji among different age groups. Conducting interviews among healthcare workers could also provide valuable information about other challenges related to smoking among adults.

## CONCLUSIONS

The results of this study highlight some important factors that affect initiation of smoking and smoking cessation among adult smokers in Fiji. These factors are categorized as individual, social, cultural and financial determinants that form a framework to combat smoking. They should be considered within the context of a culturally diverse society in Fiji. Policy makers and decision makers should consider social norms, along with cultural and religious factors in future smoking cessation programs. The factors identified in this study can be used in developing tailored interventions that affect smoking cessation. Using different health promotion theories and models such as the Behavioral Intention Model (BIM) or Health Belief Model (HBM), can increase the effectiveness of smoking cessation programs at the individual level.

## Data Availability

The data supporting this research are available from the authors on reasonable request.
